# Bioactive Compounds: Applications and Benefits for Human Health

**DOI:** 10.3390/molecules31122096

**Published:** 2026-06-15

**Authors:** Sara Baldelli

**Affiliations:** 1 Department for the Promotion of Human Science and Quality, Life San Raffaele Open University, Rome, Via di Val Cannuta, 247, 00166 Rome, Italy; sara.baldelli@uniroma5.it; 2 IRCCS San Raffaele Roma, 00166 Rome, Italy

## 1. Introduction

Scientific interest in bioactive compounds has undergone a remarkable evolution. These molecules, widely distributed in key foods such as fruits, vegetables, olive oil, spices, and cereals, but also derived from animal, microbial, or chemically synthesized sources, represent fundamental pillars of health [1]. Thanks to their strong antioxidant and anti-inflammatory properties, molecular classes such as polyphenols, terpenoids, alkaloids, and glucosinolates have demonstrated the ability to modulate crucial biochemical pathways, offering promising protective and therapeutic strategies against cardiovascular, oncological, neurodegenerative diseases, infections, and sarcopenia [2].

This Special Issue was created with the aim of gathering cutting-edge research on the isolation, characterization, and preclinical and clinical effects of these substances. With the published scientific contributions, the volume now offers an excellent overview ranging from extraction techniques to molecular mechanisms of action, confirming the cutting-edge role of research in this field.

## 2. Coverage of Emerging Trends

The contributions published in this Special Issue redefine and expand the frontiers of phytocomplexes and natural compounds applied to human health, offering new technological and mechanistic perspectives. This is paved by an in-depth review by Tomczyk and Dżugan [3], which focuses on gemmotherapy, a branch of phytotherapy that uses extracts derived from developing plant meristematic tissues, such as buds, young shoots, and shoots. Thanks to their metabolic activity, these tissues represent an incredibly concentrated source of crucial molecules, including polyphenols, phytohormones, amino acids, vitamins, and enzymes. The authors highlight how the unique phytochemical profile of bud extracts, combined with the synergistic interactions established within the phytocomplex, confers on these matrices enhanced and superior biological activity compared to that of adult plants. The study examines in detail both traditional extraction methods, such as traditional glyceric maceration, and modern, sustainable technologies, including pulsed ultrasound-assisted extraction (PUAE), analyzing the critical factors that influence the quality and phytochemical variability of the final product. The collected scientific evidence attributes a broad spectrum of bioactivities to these preparations, ranging from antioxidant and anti-inflammatory effects to immunomodulatory, antimicrobial, and anticancer properties. These characteristics suggest their promising use as supportive agents in the management of chronic inflammatory diseases, metabolic disorders, and infections, as well as innovative ingredients for functional foods and natural cosmetics.

Continuing the analysis of the molecular and nutritional mechanisms linking bioactive compounds to human pathophysiology, the review by Wróblewska et al. [4] addresses a global health issue: anemia and its correlation with *Helicobacter pylori* infection. The authors provide a clear biochemical picture of how this pathogen can profoundly alter the gastric microenvironment, triggering chronic inflammation and an increase in pH that compromise the homeostasis of essential micronutrients. In this context, the work focuses on the crucial role of vitamin C as a key bioactive molecule. Under physiological conditions, ascorbic acid present in gastric juice enhances the bioavailability of non-heme iron, reducing the ferric ion (Fe^3+^) to the ferrous form (Fe^2+^), which is significantly more absorbable at the intestinal level. *H. pylori* infection, however, causes a drastic reduction in vitamin C concentrations in the gastric lumen, drastically limiting iron absorption. At the same time, bacterial-induced hypochlorhydria hinders the proper release of vitamin B12 from dietary proteins, exacerbating the risk of micronutrient deficiencies. This systematic review not only clarifies the pathogenic pathways of gastric dysfunction-related anemia but also highlights the clinical importance of antioxidant compounds in maintaining the proper function of ion transporters and nutrient absorption.

An equally crucial line of research for this Special Issue concerns the protective efficacy of bioactive compounds in counteracting systemic oxidative stress and distal organ damage triggered by acute events. This is the context of the experimental study conducted by Erbatur et al. [5], which investigated the therapeutic potential of taxifolin, a flavonoid widely known for its strong antioxidant properties, in mitigating acute lung injury induced by hepatic ischemia-reperfusion (I/R). Using an *in vivo* model in Wistar rats, the authors demonstrated how hepatic ischemic injury generates a severe systemic inflammatory and oxidative response, capable of compromising lung function. This condition was documented by a surge in serum levels of malondialdehyde (MDA), interleukin-6 (IL-6), and total oxidant status (TOS), as well as marked histopathological deterioration of lung tissue. Pretreatment with taxifolin demonstrated a significant protective effect, halving the histological damage score, restoring total antioxidant status (TAS), and significantly reducing inflammatory markers. From a mechanistic perspective, the research uncovered the key role of the high-mobility group box-1 (HMGB1) signaling axis and the transcription factor NF-κB. Immunohistochemical analysis revealed a strong positive correlation between the expression of these two macromolecules, both of which were drastically downregulated by the administration of the flavonoid. Complementing the biological data, bioinformatic pathway analysis and molecular docking studies confirmed taxifolin’s ability to bind tightly to TLR4 receptors and the p65 subunit of NF-κB. By blocking the upstream HMGB1–TLR4–NF-κB cascade, taxifolin emerges as a promising preclinical therapeutic agent for the prevention of systemic and organ complications secondary to ischemic insults.

The work published by Mikołajczak and Nowicka [6] moves from the analysis of systemic nutritional strategies to a highly concrete biotechnological and formulation application: the development of innovative functional foods, specifically nutrient-dense health “shots.” Recognizing the growing popularity of these commercial formats due to their convenience, the authors designed and characterized formulations enriched with herbal infusions, focusing not only on the initial phytochemical profile but also on the actual bioaccessibility and bioavailability of the active ingredients. The base matrix, consisting of pear and flowering quince juice, was supplemented with various herbal blends and subjected to rigorous three-stage in vitro digestion (oral, gastric, and intestinal). The chemical and physical results favored the mint and nettle formulation for its highest total polyphenol content (579 mg/100 mL), while the shot enriched with white mulberry and yarrow stood out for its mineral content (28 mg/100 mL). In addition to their strong antioxidant properties, the developed products demonstrated strong enzyme inhibition of pancreatic lipase and lipoxygenase, suggesting potential adjuvants in the management of lipid metabolism and inflammatory conditions. The most scientifically significant finding lies in the synergistic effect of the phytocomplex: the study demonstrates that the inclusion of selected herbs, particularly those rich in rosmarinic acid, significantly improves the bioaccessibility of the active ingredients, and that the presence of menthol can further enhance their intestinal absorption. This work highlights how the rational combination of fruit matrices and botanical extracts allows for the modulation of the sensory and health properties of the finished product, offering concrete answers to the needs of modern nutraceuticals.

Dong et al. [7] shift the focus to translational pharmacology and biopharmaceutical strategies needed to overcome the intrinsic limitations of some promising natural molecules, focusing specifically on asiatic acid. This compound, an ursanic pentacyclic triterpenoid extracted from the plant *Centella asiatica* (Umbelliferae), boasts an extraordinarily broad spectrum of pharmacological activities validated by the literature, including antitumor, anti-inflammatory, hypoglycemic, antimicrobial, neuroprotective, and wound healing properties. Asiatic acid is already being used clinically in the form of tablets, capsules, and ointments, primarily for the treatment of inflammatory conditions, burns, keloids, and skin disorders. However, the authors highlight how its poor aqueous solubility, rapid first-pass metabolism, and resulting low oral bioavailability represent a severe barrier to its systemic use against other complex diseases. To address this bottleneck, the review critically analyzes strategies aimed at optimizing its chemical and physical properties. The work comprehensively maps both attempts to structurally modify the molecule and, above all, the application of cutting-edge pharmaceutical technologies. Great emphasis is placed on the development of innovative drug delivery systems, such as nanoformulations and advanced hydrogels, which have been shown to increase solubility, protect the active ingredient from premature degradation, and enhance its therapeutic efficacy. This review thus provides a solid theoretical foundation and bioengineering rationale for accelerating the clinical translation of asiatic acid and the development of future drug derivatives.

Remaining closely rooted in the topic of senescence and longevity, but shifting the focus to in vivo models and transcriptomics, the work by Qi Chen et al. [8] investigates the molecular mechanisms of *Panax ginseng*, one of the most celebrated botanicals in traditional medicine for its invigorating and pro-longevity properties. Despite its millennia-old fame, the exact phytochemical profile of its active fractions and the related biochemical pathways had not yet been fully elucidated. To address this gap, the authors used the nematode *Caenorhabditis elegans* as a model organism, evaluating various ginsenoid compounds based on parameters such as lifespan extension, resistance to environmental stress, and maintenance of physiological functions. This phenotypic screening identified ginsenoside Re as the molecule with the most pronounced anti-aging activity. The mechanistic core of the study was subsequently unraveled by combining transcriptomic techniques and RT-qPCR. Bioinformatic analysis demonstrated that differentially expressed genes (DEGs) induced by ginsenoside Re treatment are predominantly concentrated in the insulin/IGF-1 signaling (IIS) pathway, neuropeptide pathways, and lipid metabolism. From a genetic perspective, the study documented effective upregulation of key factors for longevity and stress response (including daf-16, sgk-1, skn-1, hsf-1, hsp-16.2, and sod-3) and, concurrently, significant downregulation of critical nodes of the IIS pathway, such as daf-2, age-1, and akt-2. The data confirm that ginsenoside Re exerts its geroprotective effect by finely modulating the IIS axis and the lipid profile, offering a solid scientific rationale for the use of ginseng as a targeted treatment against senile decline.

The topic of hepatoprotection is explored and addressed from a therapeutic perspective in a narrative review by Scarlata et al. [9]. The paper addresses the management of chronic liver diseases, including hepatic steatosis, fibrosis, and cirrhosis, which represent enormous global health challenges due to the limited pharmacological options currently available. In this clinical setting, the authors highlight flavonoids, a class of polyphenolic compounds widely distributed in plant sources and a cornerstone of the Mediterranean diet, focusing on the preventive and therapeutic potential of three specific molecules. The first is baicalein, extracted from *Scutellaria baicalensis*, which stands out for its ability to exert direct hepatoprotective effects by attenuating oxidative stress, inhibiting fibrogenesis processes, and positively modulating intrahepatic lipid metabolism. This is complemented by galangin, a flavonoid derived from *Alpinia officinarum* that has been widely demonstrated to have strong anti-inflammatory and anti-fibrotic properties, useful in blocking the cellular transition to chronic organ damage. Finally, the study examines isorhamnetin, a methylated flavonoid found in various fruits and medicinal herbs, characterized by evident hepatoprotective properties capable of mitigating inflammation and reducing the free radical load typical of these diseases. The review clearly outlines how the biochemical versatility of these three natural molecules offers a complementary avenue of action for the management of chronic liver disease, while also providing specific insights to guide future clinical research.

The contribution by Choi et al. [10] introduces us to the field of pathological angiogenesis, a key biological process driven by vascular endothelial growth factor (VEGF) and its receptor, VEGFR, closely linked to tumor progression. Their research focused on characterizing the anti-angiogenic effects of Lucknolide A (LA), a compound derived from the marine genus *Streptomyces*, evaluating its potential as a VEGFR2 inhibitor. Through targeted assays, the authors demonstrated that Lucknolide A selectively inhibits the proliferation of human endothelial cells, while showing minimal effects on normal fibroblasts and various tumor cell lines. From a cellular perspective, the molecule induces cell cycle arrest in S phase and promotes apoptosis by modulating key markers such as p53, Bax, and p21, and reducing the expression of the anti-apoptotic protein Bcl-2; effects that are further enhanced by VEGF stimulation. At the molecular level, treatment with Lucknolide A effectively suppresses VEGFR2 phosphorylation and the downstream signaling cascade, which includes the Akt/mTOR/p70S6K, MEK/ERK, Src, FAK, and p38 MAPK pathways, all crucial biochemical elements for endothelial survival and the genesis of new vessels. Molecular docking studies have also revealed that the compound binds to both the inactive and active conformations of VEGFR2, showing a significantly stronger affinity for the active state, suggesting its action as a kinase inhibitor. Functionally, the study documents how Lucnolide A inhibits VEGF-induced endothelial migration, tubule formation, and microvascular sprouting in both in vitro and ex vivo models. Finally, by reducing tubule formation associated with human breast cancer cells, this research suggests Lucnolide A as a promising, selective anti-angiogenic agent with concrete therapeutic potential in the treatment of cancer and other angiogenesis-dependent diseases.

Puchalski et al. [11] brings us back to the field of immunomodulation and the fight against respiratory viral infections, focusing on *Echinacea purpurea*. This perennial medicinal herb is widely known for its strong anti-inflammatory and immunomodulatory properties, traditionally exploited to relieve the symptoms of colds and flu. Although the plant contains several classes of active secondary metabolites, including caffeic acid derivatives, polysaccharides, flavonoids, glycoproteins, and alkylamides, the specific molecular targets and mechanisms of action of the individual compounds had not yet been fully elucidated in the scientific literature. This study aimed to fill this gap by testing the antiviral activity and cytokine-regulating capacity of several alkylamides predominantly found in Echinacea root extracts. The research thus led to the isolation of a specific alkylamide, Dodeca-2E,4E-dienoic acid isobutylamide, which demonstrated potent antiviral activity against both rhinovirus, the main causative agent of the common cold, and influenza virus. In addition to its direct therapeutic effect on pathogens, this specific compound exhibited a marked ability to inhibit the production of interleukin-8 (IL-8), a pro-inflammatory cytokine directly responsible for much of the reactive upper respiratory tract symptomatology and typically upregulated during infections. The broad spectrum of activity observed, combined with very low cellular cytotoxicity, positions this specific alkylamide as a promising candidate for the development of targeted and standardized treatments against the most common respiratory viral infections.

Finally, the work of Wojtunik-Kulesza et al. [12] shifts the focus of research towards neuroprotection and the analysis of the stereochemistry of natural compounds. The study focuses on carvone, a natural monoterpene whose two enantiomers, the R-(–) form and the S-(+) form, exhibit clearly distinct biological activities and organoleptic properties. The authors specifically investigated the anti-neurodegenerative potential of the S-(+)-carvone enantiomer, characterized by its typical cumin aroma, combining a rigorous experimental approach structured across *in vitro*, *in vivo*, and *ex vivo* phases. The results highlight the marked pharmacological multidirectionality of this molecule. In *in vitro* tests, S-(+)-carvone demonstrated a significant ability to inhibit the enzyme butyrylcholinesterase, a key biochemical target in the management of neurodegenerative diseases such as Alzheimer’s. At selective concentrations, the monoterpene exhibited clear neuroprotective and hepatoprotective effects, counteracting the generation of ROS and lipid peroxidation processes, while confirming the absence of hepatotoxicity. In *in vivo* tests on murine models, repeated administration of the compound at a dose of 100 mg/kg produced a significantly positive impact on memory acquisition processes, without altering general locomotor activity. The most significant translational data was finally obtained through gas chromatography-mass spectrometry (GC-MS) analysis of *ex vivo* extracted tissues: the study unequivocally demonstrated that S-(+)-carvone is able to effectively cross the blood-brain barrier and selectively accumulate within the hippocampus, the primary brain area responsible for cognitive and mnemonic functions, thus laying the foundations for future developments in the field of phytotherapy applied to neuroscience.

## 3. Conclusions

In conclusion, the eleven contributions collected in this Special Issue outline a detailed and multidisciplinary map of the role of bioactive compounds in modern medicine and functional nutrition. From the optimization of extraction and biotechnological techniques to an intimate understanding of the molecular pathways governing inflammation, oxidative stress, immunomodulation, organ protection, and cognitive decline, these studies demonstrate the immense preventive and therapeutic potential of natural matrices. The evidence presented not only confirms the scientific validity of many traditional phytochemicals but also paves the way for future challenges in the field, focused on analytical standardization, the development of advanced delivery systems, and clinical validation, necessary for the full integration of these molecules into evidence-based medicine.

A second edition of this special issue is currently open, with a submission deadline of 31 October 2026, and is available at: https://www.mdpi.com/journal/molecules/special_issues/BPK099MS01 (accessed on 9 June 2026).
